# Editorial: “One Health” approach for revealing reservoirs and transmission of antimicrobial resistance, volume II

**DOI:** 10.3389/fmicb.2023.1170407

**Published:** 2023-03-06

**Authors:** Ziad Daoud, Jean-Marc Rolain

**Affiliations:** ^1^College of Medicine, Central Michigan University, Mount Pleasant, MI, United States; ^2^Department of Clinical Microbiology & Infection Prevention, Michigan Health Clinics, Saginaw, MI, United States; ^3^IHU Mediterranee Infection, Marseille, France

**Keywords:** MDR, One Health (OH)—approach, economic impact, global change, bacterial resis

One Health emphasizes the interdependence of human, animal, and environmental health, and highlights the need for collaboration and coordinated approaches to promote the health and wellbeing of all. Published literature collectively demonstrate the importance of a One Health approach in addressing complex global health challenges, including emerging infectious diseases, zoonotic disease control, climate change, and economic benefits (Behravesh, 2016; Cunningham et al., 2017).

This Research Topic, ““*One Health” approach for revealing reservoirs and transmission of antimicrobial resistance, volume II*,” encompasses 21 manuscripts exploring antimicrobial resistance (AMR) from a One Health perspective, and investigating AMR prevalence and characteristics in different contexts, including pets, wildlife, and poultry production. The studies highlighted the potential role of animals in the transmission of AMR and emphasized the importance of surveillance and preventative measures to combat the spread of resistant bacteria. The findings underscored the interdependence of human, animal, and environmental health, and the need for a One Health approach to combat the global threat of AMR. These studies emphasized the importance of basic hygiene measures, preventative measures, and action to mitigate the spread of AMR.

In this volume II, several studies addressed the “One Health” issue in animal farms, highlighting the need for surveillance to address the public health importance of antimicrobial-resistant pathogens and their resistance mechanisms. These studies revealed the varying levels of resistant bacteria in livestock and emphasized the urgent need for AMR surveillance in various regions to identify primary risk points and the importance of monitoring antimicrobial resistance in poultry production.

A longitudinal study by Tello et al. was conducted on five dairy cattle farms to assess the dynamics of ESBL-/AmpC-/carbapenemase-producing *Escherichia coli* and their resistance profiles, along with genes responsible for resistance. Cefotaxime-resistant *E. coli* was detected in all farms, but their isolation frequency and resistance profiles varied among farms and age groups. Whole-genome sequencing of a selection of isolates recovered from two farms showed the predominance of a few genomic subtypes in one farm, while great variability of strains was observed in the other. The study highlighted the need for One Health surveillance due to the public health importance of ESBL-producing *E. coli* as pathogens and vectors for resistance mechanisms. Sellera et al. found a carbapenem-resistant NDM-1-positive *E. coli* strain (BA01) in a stranded pygmy sperm whale on the southern coast of Brazil during COVID-19 lockdown. BA01 strain belonged to the global sequence type (ST) 162 and carried blaNDM-1, in addition to other antimicrobial resistance genes and genes associated with resistance to heavy metals, biocides, and glyphosate. The strain also exhibited halophilic behavior, and *in silico* analysis confirmed the presence of halotolerance-associated genes katE and nhaA. The phylogenomics analysis clustered BA01 with poultry- and human-associated ST162 lineages circulating in European and Asian countries, and important virulence genes were detected. A study from China by Xiaoshen Li, Xie, et al. reports that livestock-associated methicillin-resistant *Staphylococcus aureus* (LA-MRSA) is present in high frequencies in samples from swine, with spa type t571 accounting for a higher proportion. MRSA ST398 strains may spread among swine, humans, and the environment, and there is potential transmission among different countries. The MRSA isolates showed multidrug-resistant phenotypes, and the poxtA-carrying segment and the coexistence of cfr and optrA in a plasmid were detected in MRSA ST398 for the first time. The study highlighted the urgent need for LA-MRSA surveillance, given the potential transmission among humans, animals, and the environment. Another study from China by Tang et al. looked into the prevalence of antimicrobial resistance (AMR) and the *mcr-1* gene in poultry-derived *E. coli* in Qinghai Plateau is investigated. Three hundred forty-six *E. coli* strains were isolated, with eight carrying *mcr-1*, and multidrug-resistant strains accounted for 95.66% of the total isolates. Colistin resistance was identified in 12 *E. coli* strains. Whole-genome sequencing revealed 46 AMR genes and 36 virulence factors. The *mcr-1* gene was located on various plasmids and its spread through the food chain is a concern. The study emphasized the need for strengthened AMR surveillance in various regions under the background of one health.

Temmerman et al. investigated the impact of fluoroquinolone antibiotics on the microbiome and resistome of broiler chickens receiving an optimized dose of enrofloxacin with or without synbiotic supplementation. Results showed that the optimized dose caused significant perturbations in the microbiota and increased the antibiotic resistance gene (ARG) resistome diversity. Symbiotic treatment reduced the diversity and number of enriched ARGs, suggesting a positive impact on the gut resistome. Proteobacteria were significantly increased in the cecal metagenome of chickens receiving enrofloxacin, and the optimized dosage application resulted in a two-fold higher number of affected ARG compared to high dosage application. These findings highlight the need to understand the impact of antibiotic use on the gut resistome of poultry and to develop strategies to mitigate potential resistance gene spread. In another study by Zaidi et al. study examined *Enterococcus hirae* isolates, primarily from beef production systems, for antimicrobial resistance and comparative genomics. The isolates showed high resistance to tetracycline and erythromycin, with multi-drug resistance present in over 50% of the beef production isolates. Genes for tetracycline and macrolide resistance were frequently found in these isolates. The resistance profiles appeared to reflect the kind of antimicrobial usage in beef and human sectors. Comparative genomic analysis showed *E. hirae* has unique genes associated with vitamin production and cellulose and pectin degradation, which may support its adaptation to the bovine digestive tract. *Enterococcus faecium* and *Enterococcus faecalis* more frequently harbored virulence genes, suggesting niche specificity within these species. Menck-Costa et al. monitored antimicrobial resistance in broiler chicken farms over a 2-year period in northern Paraná, Brazil, with a focus on evaluating resistance to fosfomycin and β-lactams. *Escherichia coli* was isolated from various sources, including cloacal swabs, meconium, poultry feed, water, poultry litter, and *Alphitobius diaperinus*. The results showed a high frequency of multidrug-resistant *E. coli*, with 51% of strains being ESBL-positive, and the *blaCTX-M-1* group being the most common. The fosA3 gene was also prevalent, particularly in day-old chickens. The study highlights the need for longitudinal monitoring of antimicrobial resistance in poultry production to identify primary risk points.

Other four studies in this volume explored the prevalence and characteristics of antibiotic-resistant bacteria in wildlife in different regions. The studies found high resistance rates to various antibiotics, including colistin and third-generation-cephalosporin, as well as the presence of new sequence types and mobile genetic elements carrying AMR genes. These studies highlight the potential role of wildlife in the transmission of AMR and emphasize the need for further research, effective surveillance, and strict biosecurity strategies to prevent the spread of antibiotic-resistant bacteria and monitor the emergence of mobile genetic elements and integrons. These findings underscore the importance of the One Health approach to address the issue of AMR, recognizing the interdependence of human, animal, and environmental health. Abdallah et al. investigated the prevalence and dynamics of antibiotic-resistant Enterobacterales in wildlife in antibiotic-limited areas of Senegal. Fecal samples were collected from monkeys and apes, and non-fecal samples were collected in 2015 and 2019. The study found a high resistance rate to colistin and third-generation-cephalosporin, and 46% of isolates belong to 34 new sequence types. The presence of a transposon that carries AMR genes is intriguing since no antibiotics are used in the non-human primates studied. The results highlight the potential role of wildlife in the transmission of AMR and the need for further research to better understand this risk. Mercato et al. investigated the prevalence and characteristics of Extended Spectrum Beta-Lactamases (ESβLs) producing *E. coli* strains in wild boars in Northern Italy. The analysis of 60 animals revealed a prevalence of 23.3% for ESβLs-producing *E. coli* strains, which were found to be multi-drug resistant and carried different types of plasmid replicons. Genome analysis showed the presence of two pandemic sequence types, ST131 and ST10, with different collections of virulence factors, and suggested the potential for AMR gene exchange. This highlights the importance of including wild boars in surveillance programs and understanding their role in the spread of AM resistance in the “One Health” framework. Li, Cheng, et al. investigated the mechanisms of chromosome-mediated and plasmid-mediated colistin resistance in *E. coli* isolates from animals in Sichuan Province, China. Out of 101 colistin-resistant isolates, the MCR gene was detected in 58 of them. The study found that the *MCR-1* gene was primarily localized on an *IncX4*-type and IncI2 plasmid. Furthermore, colistin resistance in MCR-negative isolates was related to chromosomal point mutations including the two-component systems PhoP/PhoQ and PmrA/PmrB and their regulators MgrB. The study highlights the high prevalence of colistin resistance in farms in Sichuan, China, and the importance of monitoring and controlling the spread of antibiotic resistance. A study conducted by Yan et al. on giant pandas found a high prevalence of multi-drug-resistant (MDR) *Klebsiella pneumoniae* (*K. pneumoniae*) in their feces. Antimicrobial susceptibility testing results showed that 16.5% of *K. pneumoniae* isolates were MDR, mainly resistant to AMX, DOX, CHL, SXT, and TMP. The study also found 50 different types of ARGs and 13 mobile genetic elements. Furthermore, the investigation of integrons revealed the emergence of class 1 integrons in 96.67% of the isolates, while class 2 and class 3 integrons were not screened. The study suggests the need for effective surveillance and strict biosecurity strategies to prevent the spread of MDR bacteria and monitor the emergence of mobile genetic elements and integrons.

As per the wide spread of resistance genes, four articles covered different aspects of antimicrobial resistance (AMR) from a One Health perspective, including identifying drug resistance mechanisms, exploring the role of mobile genetic elements in spreading AMR genes, investigating potential treatments, and highlighting the threat of mobile tigecycline resistance. They emphasize the urgent need for action and One Health approaches to combat this global threat. The study by Zeng et al. aimed to identify the species of a carbapenem-resistant *Pseudomonas* strain and analyze its integrative and conjugative element formation mechanism. Single molecule real-time sequencing was used to identify the species as *Pseudomonas juntendi* and determine its drug resistance mechanism. The study found that the capture of the accA4' gene cassette by the Tn402-like type 1 integron (IntI1-blaIMP-1) forms In1886 before being captured by the ΔTn4662a-carrying ICE 1276, which confers carbapenem resistance to *P. juntendi* 18091276. In this same context, a review by Algarni et al. discussed the role of mobile genetic elements (MGEs) in the spread of antimicrobial resistance (AMR) genes in *Salmonella enterica* and related enteric bacteria. The mobilome, which includes all mobile genetic elements that can spread genes, played a key role in the rapid spread of AMR genes in *S. enterica*. The review highlights the importance of studying the dynamics of AMR genes in the mobilome for the identification and verification of emerging multidrug resistance. Abdelraheem et al. investigated the antibacterial and anti-biofilm effects of vitamin C (ascorbic acid) on *Pseudomonas aeruginosa*. The minimal inhibitory concentration of ascorbic acid and antibiotics was determined, and the effect of ascorbic acid on biofilm-forming isolates was assessed. Real-time PCR was used to test the effect of ascorbic acid on antibiotic-resistant and biofilm encoding genes. *In vivo*, rats were infected with *P. aeruginosa* and treated with ascorbic acid alone or combined with an antibiotic. Ascorbic acid had a 100% biofilm inhibitory effect at sub-inhibitory concentrations and downregulated genes related to biofilm formation and antibiotic resistance. *In vivo*, combining ascorbic acid and ceftazidime had a synergistic effect. The study suggests that vitamin C could be prescribed with antibiotics for the treatment of bacterial infections in clinical settings. The threat of mobile tigecycline resistance (MTR) to the clinical efficacy of the antibiotic tigecycline, which is used to treat deadly infections caused by superbugs was discussed by Anyanwu et al. MTR is caused by plasmid-mediated transmissible genes that confer high-level resistance to tigecycline. The article stressed the need for urgent global intervention to mitigate the spread of MTR, which has significant health and economic impacts due to limited options for therapy. The article covered the antimicrobial activity of tigecycline, mechanism of tigecycline resistance, distribution, reservoirs, and traits of MTR gene-harboring isolates, causes of MTR development, possible MTR gene transfer modes and One Health implications, and MTR spread and mitigating strategies.

Three studies revealed the potential role of pets as a reservoir of antimicrobial-resistant bacteria or genes for humans, the inter-human transmission of LA-MRSA, and the high prevalence of fecal colonization with ESBL-Ec among children < 1-year-old in rural households in Bangladesh. The studies emphasized the importance of basic hygiene measures, surveillance, and preventative measures to combat the spread of antimicrobial resistance. Gruel et al. found that 7.6% of household and shelter pets carry extended-spectrum beta-lactamase-producing Enterobacteriaceae (ESBL-E), with the only risk factor being a stay in a shelter. The study identified the presence of a *blaCTX-M-1*/IncI1-Iγ/sequence type (ST3) plasmid in 11 of the ESBL-producing *E. coli* isolates, and noted the potential role of pets as a reservoir of antimicrobial-resistant bacteria or genes for humans, highlighting the importance of basic hygiene measures by owners of companion animals. Similarly, Konstantinovski et al. assessed the genetic epidemiology and clinical characteristics of LA-MRSA in an urban area with limited livestock. LA-MRSA strains were cultured from 81 patients, and most patients had no livestock link. Contact tracing led to the identification of two hospital transmissions and five additional clusters without a known epidemiological link. The study confirms that LA-MRSA may cause a relevant burden of disease in urban areas, suggesting inter-human transmission. The presence of virulence genes warrants future surveillance and preventative measures. A study by Badrul Amin et al. conducted in rural households in Bangladesh found a high prevalence of fecal colonization with extended spectrum β-lactamase-producing *E. coli* (ESBL-Ec) among children < 1-year-old. The study aimed to identify the sources of ESBL-Ec colonization in children by analyzing *E. coli* isolates from child stool, child's mother stool, and point-of-use drinking water. The study found that ESBL-Ec colonization in children is linked to the colonization status of mothers and exposure to household environments contaminated with ESBL-Ec. Interventions such as improved hygiene practices and safe drinking water supply may help reduce the transmission of ESBL-Ec at the household level.

The last three studies explore the issue of antimicrobial resistance (AMR) from a One Health perspective. The studies investigate the prevalence and mechanisms of AMR in different contexts, such as household pets, urban areas, rural households, and environmental sources. The study by Aslam et al. collected 775 samples from human, animal, and environmental sources and found *K. pneumoniae* in 15.7% of them, with the highest prevalence in humans. The isolates showed significant resistance to antibiotics, except colistin. The study identified various antibiotic resistance genes, with the highest prevalence of *blaCTX-M*. Multi-locus sequence typing analysis revealed 21 distinct sequence types and 13 clonal complexes. The distribution of multi-drug resistant *K. pneumoniae* clones in the community and associated environment is alarming for public and animal health, requiring attention from health policymakers. Lawther et al. used the ResFinder database to identify resistance genes in over 5,800 metagenomes. Antimicrobial resistance genes were diverse and widespread, with tetracycline resistance genes being the most prevalent in livestock microbiomes. The oleandomycin resistance gene was the most abundant gene in soil. Fifty-five resistance genes were shared by the four microbiomes, with 11 actively expressed in two or more microbiomes. The study provides a global insight into the diverse and abundant antimicrobial resistance gene reservoirs in both livestock and soil microbiomes, posing a serious threat to public health. Finally, Lawal et al. conducted a systematic review to provide an update on the clonal distribution of MRSA in Africa. Genotyping data was based primarily on multilocus sequence types (STs) and Staphylococcal Cassette Chromosome mec (SCCmec) types. MRSA with diverse spa and SCCmec types in CC5 and CC8 were reported across the continent. The study identified an increase in the distribution of ST1, ST22, and ST152, but a decline of ST239/241 in Africa. There is a need to strengthen genomic surveillance capacity based on a “One-Health” strategy to prevent and control MRSA in Africa.

To conclude, many studies are currently looking into the antimicrobial resistance in the One Health context, this is providing more data knowledge. It is very important for future studies to look at Climate change and One Health and the economics of One Health to highlight the importance of this approach in addressing the interdependent health of humans, animals, and the environment (Machalaba et al., 2017; Zinsstag et al., 2018).

## Author contributions

All authors listed have made a substantial, direct, and intellectual contribution to the work and approved it for publication.
